# Effect of the sagittal osteotomy inclination angle on the posterior tibial slope change in high tibial osteotomy: three-dimensional simulation study

**DOI:** 10.1038/s41598-022-23412-5

**Published:** 2022-11-10

**Authors:** Jai Hyun Chung, Chong Hyuk Choi, Sung-Hwan Kim, Sung-Jae Kim, Yong June Suk, Min Jung

**Affiliations:** 1 The Department of Medicine, Yonsei University Graduate School, Seoul, Republic of Korea; 2 The Department of Orthopaedic Surgery, The Arthroscopy and Joint Research Institute, Yonsei University College of Medicine, 134, Shinchon-dong, Seodaemun-gu, C.P.O. Box 8044, Seoul, 120-752 Republic of Korea; 3 The Department of Orthopaedic Surgery, Yonsei University College of Medicine, Seoul, Republic of Korea

**Keywords:** Osteoarthritis, Osteoarthritis

## Abstract

In performing medial open-wedge high tibial osteotomy, it is recommended not to alter the posterior tibial slope. However, it remains unclear whether the osteotomy inclination angle affects the posterior tibial slope in the sagittal plane. This study aimed to verify how anterior or posterior osteotomy inclination angle affects the tendency of change in the posterior tibial slope and to conduct quantitative analysis of the extent to which the posterior tibial slope changes according to the degree of the osteotomy inclination angle change in MOWHTO. Computed tomography images of 30 patients who underwent MOWHTO were collected. Three-dimensional models of preoperative original tibia were reconstructed, and virtual osteotomies were performed. The sagittal osteotomy inclination angles formed by the osteotomy line and the medial tibial plateau line were classified as positive in case of anteriorly inclined osteotomy and negative in case of posteriorly inclined osteotomy. Thirteen osteotomies were performed for each tibial model at intervals of 5° from − 30° to 30°. The posterior tibial slope was assessed, and the proportional relationship between the sagittal osteotomy inclination angle and the posterior tibial slope change was analyzed. The posterior tibial slope changed significantly after osteotomy (*p* < 0.001), except for the cases where the sagittal osteotomy inclination angles were 5°, 0°, and − 5°. Anteriorly and posteriorly inclined osteotomy caused increase and decrease in the posterior tibial slope, respectively. As the inclination angle increased by 1°, the posterior tibial slope increased by 0.079° in anterior inclination osteotomy, while in posterior inclination osteotomy, as the inclination angle decreased by 1°, the posterior tibial slope decreased by 0.067°. The osteotomy inclination angle in the sagittal plane significantly affected the posterior tibial slope. When there was an inclination angle occurred between the osteotomy line and the medial tibial plateau line in the sagittal plane, the posterior tibial slope changed after MOWHTO. The posterior tibial slope tended to increase in anteriorly inclined osteotomy and decrease in posteriorly inclined osteotomy. The change in the posterior tibial slope was proportionally related to the absolute value of the osteotomy inclination angle.

## Introduction

High tibial osteotomy is an effective procedure for medial compartment osteoarthritis of the knee with varus deformity in middle-aged patients^1–3^. It reduces medial compartment pressure of the knee joint by realigning the mechanical axis of the lower extremity from medial to lateral compartment and redistributing the joint pressure, thereby reducing the pain and improving the function^4–6^. A medial open-wedge high tibial osteotomy (MOWHTO) has become increasingly common in recent years due to several associated advantages, such as higher accuracy of correction^7^, lower risk of peroneal nerve complication compared with lateral closing wedge high tibial osteotomy^5^. The proximal tibial metaphyseal bone stock was also preserved without disruption of tibiofibular joint^8^. This maintenance of the proximal tibial configuration makes it easier to convert to total knee arthroplasty^9,10^. Previous studies have shown that MOWHTO produces good clinical results including the low pain score and high activity levels with advanced Knee injury and Osteoarthritis Outcome Score (KOOS) and The Western Ontario and McMaster Universities Arthritis Index (WOMAC) scores^2,11–13^.

MOWHTO focuses primarily on realignment of the lower extremity in the coronal plane to relieve pain associated with osteoarthritis of the medial compartment of the knee^4,5^. However, as the knee joint is a three-dimensional (3D) structure, when the osteotomy on the proximal tibia for the change of mechanical axis of the lower extremity (the line from the center of the femoral head to the center of the ankle joint^14^) in the coronal plane is performed, the structural shape of the tibia in the sagittal plane also inevitably changes. Among the changes caused by the structural shape of the tibia in the sagittal plane, the change in the posterior tibial slope has significant clinical impact as it influences the biomechanics of the tibiofemoral joint^15,16^. Increase in the posterior tibial slope can cause the tibia to shift anteriorly in relation to the femur and overload the anterior cruciate ligament and the patellofemoral joint^15,17,18^. The posterior tibial slope has been reported to change after MOWHTO^19^, and this change can be affected by various factors^20–23^. The most common cause of change in the posterior tibial slope is an improper value of osteotomy opening gap ratio according to the difference between anterior and posterior osteotomy opening gaps^20,21^. In addition to the opening gap ratio, it has been reported that the osteotomy inclination angle in the sagittal plane also affects the change in the posterior tibial slope. A previous clinical study reported that 87.1% of the osteotomy lines were inclined anteriorly in the sagittal plane, and anteriorly inclined osteotomy was positively correlated with the change in the posterior tibial slope^22^. Another clinical study^23^ demonstrated that even if the osteotomy opening gap ratio is preserved at an appropriate value during MOWHTO, the postoperative posterior tibial slope can change depending on the sagittal osteotomy inclination angle. The postoperative posterior tibial slope increased when the anterior part of the osteotomy line was inclined distally with respect to the line parallel to the medial tibial plateau line in the sagittal plane, and decreased when the anterior part of the osteotomy line was inclined proximally. The results of these studies^22,23^ suggest that the posterior tibial slope may increase or decrease depending on whether the osteotomy line is inclined anteriorly or posteriorly in the sagittal plane. However, the above-mentioned studies did not provide sufficient conclusions for quantitative analysis of the extent to which the change in the osteotomy inclination angle in the sagittal plane affects the change in the posterior tibial slope. Therefore, the present study aimed to verify how the anterior or posterior osteotomy inclination angle affects the tendency of change in the posterior tibial slope and to conduct a quantitative analysis to determine the extent of change in the posterior tibial slope according to the degree of change in the osteotomy inclination angle. This study was based on the hypothesis that, with an increase in the inclination angle between the sagittal osteotomy line and the medial tibial plateau line, the degree of change in the posterior tibial slope after MOWHTO would also increase.

## Methods

### Patients

Patients who underwent MOWHTO from November 2014 to November 2017 at our hospital were retrospectively reviewed after approval from the institutional review board of our institution. This study received exemption from informed consent by the institutional review board. Research process was performed in accordance with the Declaration of Helsinki. The inclusion criteria were as follows: (1) symptomatic osteoarthritis in medial compartment treated with MOWHTO, (2) varus alignment of the lower extremity (hip-knee-ankle angle > 5°), (3) no fracture or osseous deformity of the index lower extremity other than osteotomy surgery, (4) no previous surgery around the index knee, and (5) no ligament injury of the index knee.

### 3D reconstruction of computed tomography images

**Figure 1:**
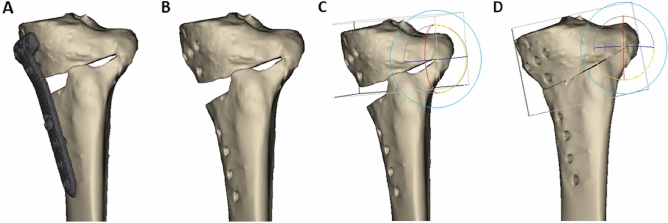
Steps to create a three-dimensional (3D) model of a pre-osteotomy original tibia. (**A**) reconstructed 3D model of tibia without soft tissue. (**B**) Virtual elimination of plate and screws from the 3D model (**C**) Rotation axis setting with the position about 1.0 cm before the lateral cortex and 1.5 cm below the articular surface as the hinge for restoration to pre-osteotomy original tibia. (**D**) Restoration to preoperative status of tibia by rotating the proximal segment around the rotation axis and eliminating the osteotomy gap. These figures were created using the Mimics software (version 17; Materialise, Leuven, Belgium).

Digital Imaging and Communications in Medicine (DICOM) data of the postoperative computed tomography (CT) images of the included patients were extracted from the picture archiving and communication system (Centricity PACS, GE Medical System Information Technologies, Milwaukee, Wisconsin, USA). A postoperative lower leg CT scan from the distal femur to the ankle joint, including the entire tibia, was conducted for all patients on the day of the surgery, with the knee in a fully extended position, to determine the postoperative status related to osteotomy surgery, such as lateral cortical hinge fracture and plate and screw position. The CT scans (Sensation 64, Siemens Healthcare, Erlangen, Germany) had following parameters: tube voltage of 120 kVp, tube current of 135–253 mA, acquisition matrix of 512 pixels × 512 pixels, scan field of view of 134 to 271 mm, and slice thickness of 0.6 to 1 mm. The extracted DICOM files were imported into Mimics software (version 17; Materialise, Leuven, Belgium). The 3D volumes of the distal femur, proximal tibia, plate and screws were reconstructed using manual thresholding. The plate and the screws (Fig. 1-A) were then subtracted from the proximal tibia (Fig. 1-B). Thereafter, the 3D tibia model was restored to its pre-osteotomy original tibia by rotating the proximal tibia segment. The location of the hinge for the rotation was the innermost part of the osteotomy, which was used when opening the osteotomy during MOWHTO. The line where the lowermost osteotomy plane of the proximal tibial segment and the uppermost osteotomy plane of the distal tibial segment intersected was set as the axis of rotation. It was located approximately 1.0 cm medial to the lateral cortex and 1.5 cm below the joint surface in the coronal plane(Fig. 1-C)^24,25^. The rotate tool in Mimics software was used to restore the original tibial model. The tibia model was arranged so that the axis of rotation could be seen as a single point, and the center of rotation in the rotate tool was positioned at that point. The pre-osteotomy original 3D tibial model was restored by rotating the proximal tibial segment until both the upper and lower osteotomy planes met, and the proximal and distal part of the medial cortex of the tibia came into contact with each other (Fig. 1-D). The amount of rotation was almost the same as the correction angle performed during each patient's operation.

### Simulation of medial open wedge high tibial osteotomy for the 3D reconstructed tibia model

**Figure 2:**
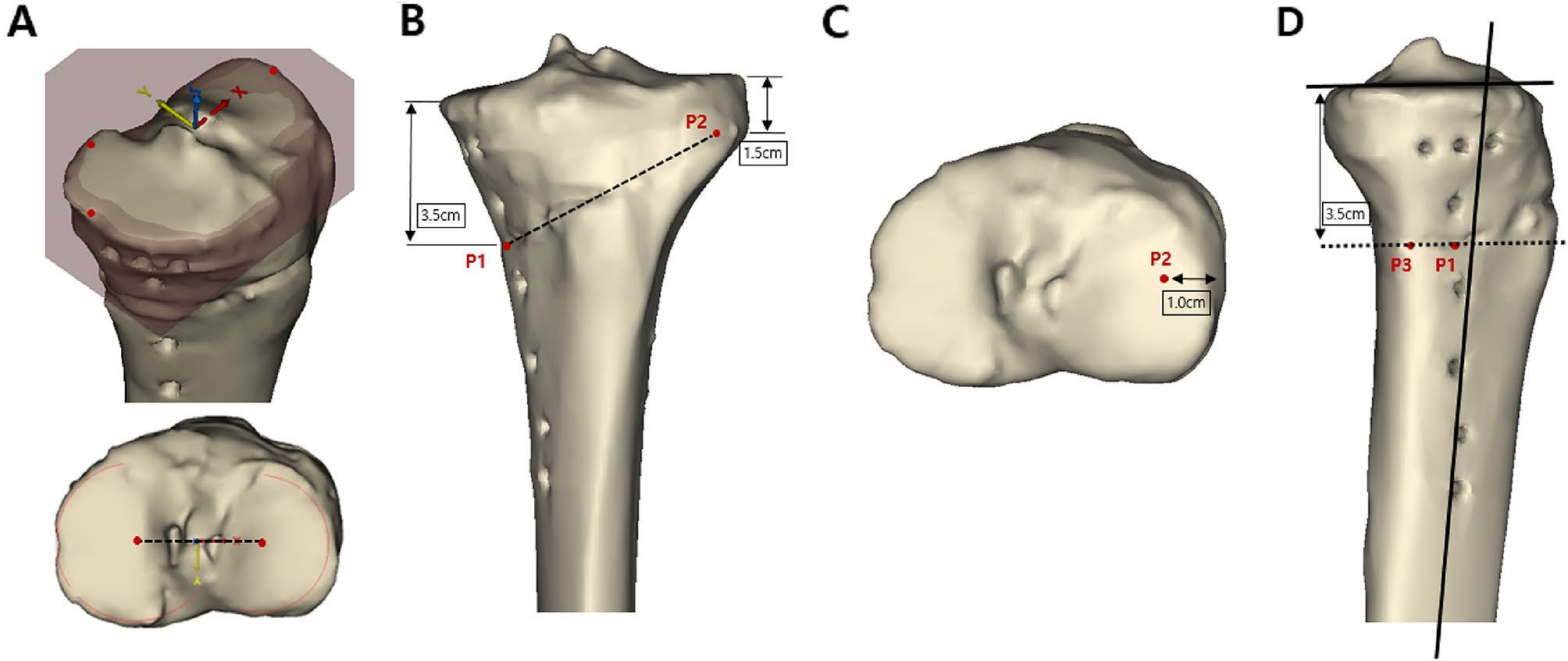
Positioning of point 1 (P1), point 2 (P2), and point 3 (P3) on the three-dimensional (3D) images of tibia. A. Determination of the joint plane and the coordinate system of proximal tibia by defining the *x*-, *y*-, and *z*-axes in the 3D model. The joint plane was defined by the 3 points (the most medial point of the medial tibial plateau, the most posterior point of the medial tibial plateau, and the most lateral point on the lateral tibial plateau). The *x-*axis was defined by the line connecting the centers of the best-fit circles of the medial and lateral plateau on the joint plane. The *y-* and *z-*axes were defined by cross-product of the x-axis B. Initially, P1, the starting point of osteotomy, was placed on the anteromedial cortex of proximal tibia, 3.5 cm below the medial end of the tibial plateau. P2, which was used as a hinge, was located 1.0 cm medially from the lateral cortex of tibia on the x-axis and 1.5 cm below the articular surface on the z-axis. C. In the axial plane, P2 was positioned at the midpoint of the entire length of anteroposterior articular surface. D. In the sagittal plane, P3 was placed at the posteromedial cortex of tibia. The angle between the line formed by P1 and P3 and the line of the medial tibial plateau was defined as the osteotomy inclination angle in the sagittal plane. These figures were created using the Mimics software (version 17; Materialise, Leuven, Belgium).

**Figure 3:**
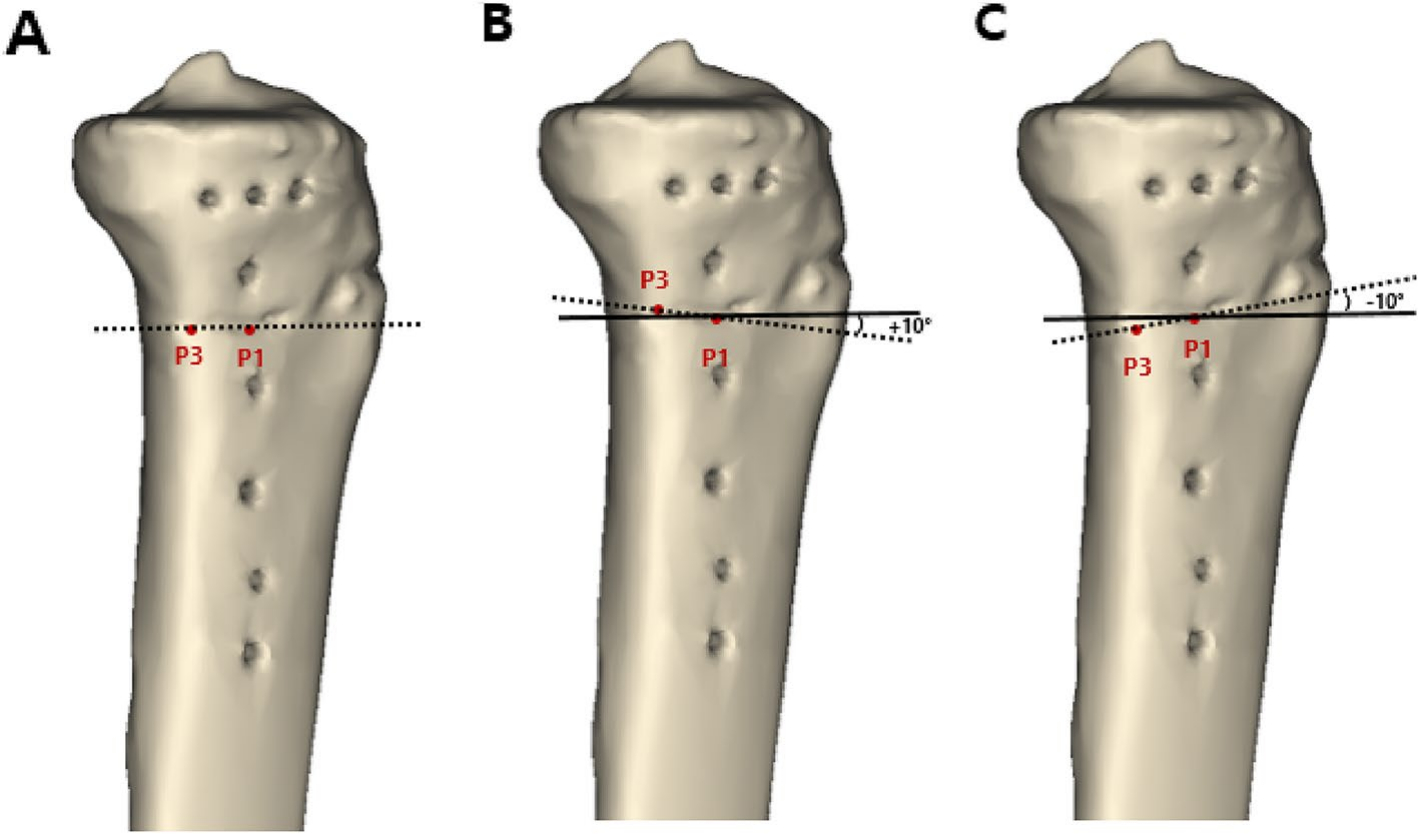
In the sagittal plane, the angle between the dotted line formed by P1 and P3 and the solid line parallel to the medial tibial plateau line was defined as the osteotomy inclination angle. (**A**) Parallel osteotomy line (0°). (**B**) Anteriorly inclined osteotomy line (10°). (**C**) Posteriorly inclined osteotomy line (− 10°). These figures were created using the Mimics software (version 17; Materialise, Leuven, Belgium).

**Figure 4:**
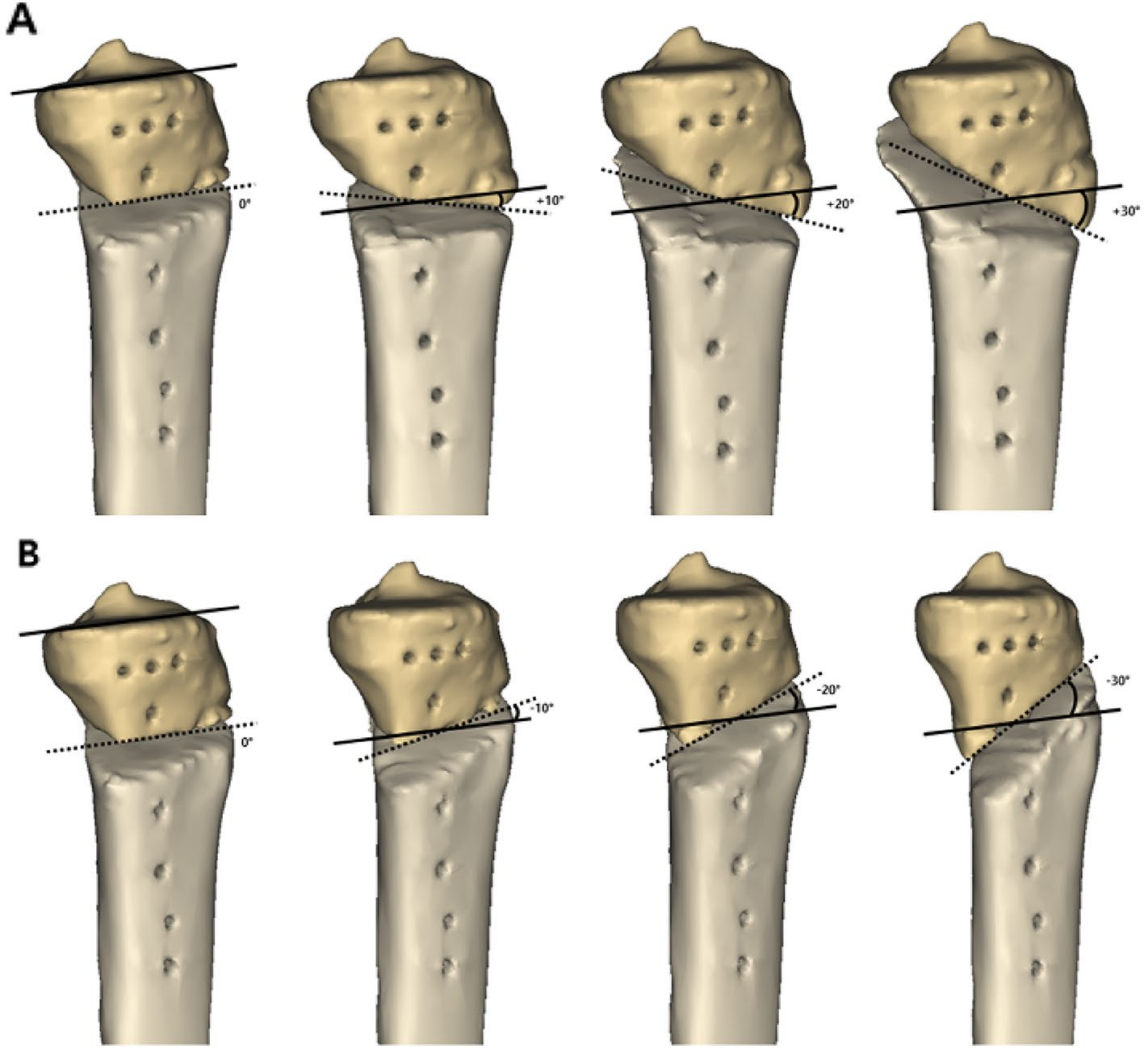
Simulation of osteotomy was performed. Thirteen osteotomy inclination angles in the sagittal plane were applied to the three-dimensional tibial model. The osteotomy inclination angle was changed from − 30° to 30° at intervals of 5° by changing the position of P3. The anterior-to-posterior osteotomy opening gap ratio was maintained at 67%. (**A**) Anteriorly inclined osteotomy (0°, 10°, 20°, 30°). (**B**) Posteriorly inclined osteotomy (0°, − 10°, − 20°, − 30°). These figures were created using the Mimics software (version 17; Materialise, Leuven, Belgium).

TO verify the effect of anterior or posterior osteotomy inclination angle on the tendency of change in the posterior tibial slope and to quantitatively analyze how much the posterior tibial slope changes according to the degree of change in the osteotomy inclination angle, osteotomy with various inclination angles in the sagittal plane was performed on the restored 3D tibia model. To perform the virtual osteotomy, the tibial coordinate system was first determined by defining the *x*-, *y*-, and *z-*axes in the 3D model. The *x*- and *y*- axes were located on the joint plane of the tibia. To clarify the conditions for creating the joint plane of the tibia, 3 points were set on the surface of the tibial plateau according to the previous study^26^. The first point is on the most medial point of the medial tibial plateau, the second one is on the most posterior point of the medial tibial plateau, and the third one is on the most lateral point on the lateral tibial plateau. These 3 points made a joint plane of tibia (Fig. 2-A). The *x*-axis was defined as the line formed by each midpoint on the surface of the medial and lateral condyles of the tibia. To define the *x*-axis on the joint plane of the tibia, the midpoint of each plateau was established by the best-fit circle method^27^. Two circles that best fit the peripheral margin of medial and lateral plateaus were drawn on the joint plane. The centers of the best-fit circles were marked as the midpoint, and the line connecting the two midpoints was defined *x*-axis. The *y*-axis was determined as the vector perpendicular to the *x*-axis in the axial plane, and the *z*-axis was determined as the vector perpendicular to the *x*-axis in the coronal plane. After defining the *x-, y-,* and *z-axes*, the true lateral position of the 3D tibial model was obtained by manipulating the femur so that the medial and lateral condyles were overlapped in the sagittal plane^28^. At the same time, the *y*-axis was positioned horizontally and parallel to the ground, in accordance with a previous study^28^. To leave only the tibial model, the femur was virtually eliminated. Following this, the coronal plane of the 3D model was obtained by rotating the model by 90° around the *z*-axis. Second, three points were determined to set the osteotomy plane. In the coronal plane, point 1 (P1), the starting point of osteotomy, was placed on the anteromedial cortex of the proximal tibia, 3.5 cm below the medial end of the tibial plateau^25^. Point 2 (P2), which was used as a hinge, was located 1.0 cm medially from the lateral cortex of the tibia on the *x*-axis, 1.5 cm below the articular surface on the *z*-axis (Fig. 2-B)^24,25,29^, and at the midpoint of the entire length of the anteroposterior articular surface on the *y*-axis (Fig. 2-C). In addition to P1, as one of the two guide points for initiating the osteotomy, point 3 (P3) was placed on the posteromedial tibial cortex in the sagittal plane. The line connecting P1 and P3 was defined as the sagittal osteotomy line (Fig. 2-D). The angle between the line formed by P1 and P3 and the line of the medial tibial plateau was defined as the osteotomy inclination angle in the sagittal plane. In order to obtain an osteotomy line parallel to the medial tibial plateau line in the sagittal plane, P3 was positioned such that the line formed by P1 and P3 was parallel to the medial tibial plateau line. By changing the position of P3, while P1 and P2 were fixed, the osteotomy inclination angle could be changed (Fig. 3). Third, osteotomy was performed virtually along the plane including P1, P2, and P3. The osteotomy gap was opened according to the correction angle applied to each patient at the time of the actual surgery as measured using the Miniaci method^30^. The osteotomy gap ratio between the anterior and posterior openings was set to 0.67, based on a previous study^21^. Various osteotomy inclination angles in the sagittal plane were applied to the 3D tibial model. The osteotomy inclination angle was changed from –30° to 30° at intervals of 5° by changing the position of P3 (Fig. 4). The angle value was classified as positive in case of an anteriorly inclined osteotomy, in which the anterior part of the osteotomy line was inclined distally compared with the line parallel to the medial tibial plateau line (Fig. 4-A). Conversely, the angle value was classified as negative in the case of posteriorly inclined osteotomy, in which the anterior part of the osteotomy line was inclined proximally (Fig. 4-B). To make an anteriorly or posteriorly inclined osteotomy, P3 was set proximal or distal to the line passing through P1 while passing parallel to the medial plateau line. A total of 13 osteotomies were performed for each 3D tibial model at intervals of 5° for inclination angle from –30° to 30°.

### Measurement of the posterior tibial slope with 3D tibial model

**Figure 5:**
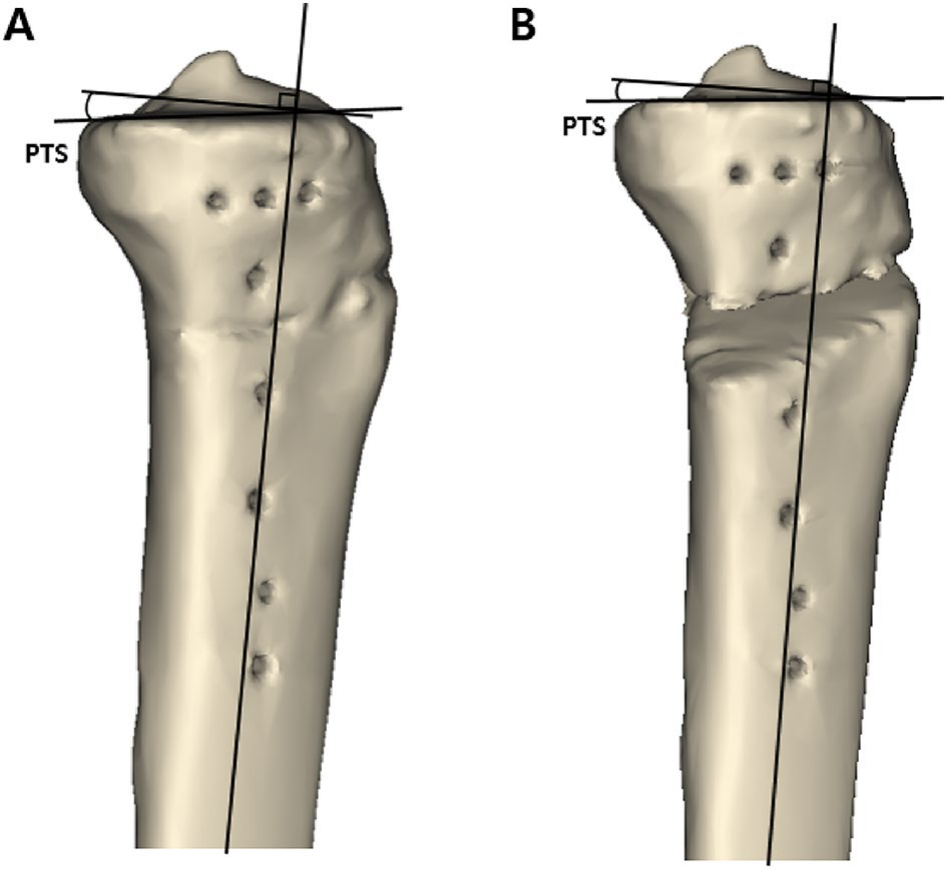
Measurement of the posterior tibial slope. In a true lateral position, the image of the 3D tibial model was captured and the posterior tibial slope was measured. The medial tibial plateau line and the line perpendicular to the line bisecting the tibial shaft were drawn. Posterior tibial slope was defined by the angle formed by these two lines. (**A**) Posterior tibial slope of the preoperative original tibia. (**B**) Posterior tibial slope of the virtually osteotomized tibia. PTS = posterior tibial slope. These figures were created using the Mimics software (version 17; Materialise, Leuven, Belgium).

The posterior tibial slope was measured on the 3D tibial model. In a true lateral position^28^, the image of the 3D tibial model was captured and measurement of the posterior tibial slope was conducted as presented in a previous study^23^. The posterior tibial slope was defined as the angle formed by the medial tibial plateau line and the line perpendicular to the line bisecting the tibial shaft (Fig. 5). For each case, the posterior tibial slopes were measured using an original tibial model that was restored to the preoperative status (Fig. 5-A) as well as 13 simulation experimental models by virtual osteotomy (Fig. 5-B).

### Statistical analysis

Repeated measured analysis of variance (ANOVA) was used to compare the postoperative posterior tibial slopes after the virtual osteotomy. The sphericity of the data was confirmed by Mauchly’s test. As the sphericity was not met, Huynh–Feldt correction was used. To make pairwise comparisons, the post hoc analysis with adjusted P-value was conducted by Bonferroni correction. A paired *t*-test was used to compare between the postoperative posterior tibial slope after virtual osteotomy with each sagittal osteotomy inclination angle and the preoperative posterior tibial slope measured in the original preoperative tibial model. A linear mixed model was used to analyze the effect of the sagittal osteotomy inclination angle on the change in the posterior tibial slope. In the linear mixed model, the Akaike information criterion (AIC) and Bayesian information criterion (BIC) were compared, and the model with a smaller AIC or BIC value was selected as the final model^31^. The coefficient (*β*) was obtained from a linear mixed model under a random intercept model with a first order autoregressive structure. The level of significance was set at *p* < 0.05. Statistical analyses were conducted using the IBM SPSS Statistics for Windows software (version 26.0; IBM, Armonk, New York, USA). The scatter plot was obtained using the R statistical software (version 3.6.2; R Foundation for Statistical Computing, Vienna, Austria).

### Ethical approval

The study was approved by the Institutional Review Board of Severance Hospital, Yonsei University College of Medicine (4-2019-0518).

### Informed consent

This study received exemption from informed consent by the Institutional Review Board.

## Results

Thirty knees from 30 consecutive cases meeting the inclusion criteria were included. The demographic data of all the patients included in this study are listed in Table 1. Nine of the 30 patients were male (30%) and 21 were female (70%), with an average age of 56.2 years (range, 43–61 years) and average body mass index of 26.9 kg/m^2^ (range, 23.2–30.0 kg/m^2^). The mean preoperative and postoperative hip-knee-ankle angles were − 6.5° (range, − 4 to − 10.9°) and 2.6° (range, 0.7–4.7°), respectively. The mean preoperative weight-bearing line ratio was 21.1% (range, 10.3–35.7%), and the mean postoperative weight-bearing line ratio was 61.1% (range, 53.1–73.0%). The mean correction angle was 10.3° (range, 6.4–16°), and the mean preoperative posterior tibial slope, measured using the restored original 3D tibial model, was 8.9° (Table 1).

**Table 1:** Demographic data of patients.

Parameter	
Age (years)^a^	56.2 ± 2.7
Sex (Male/Female)^b^	9 (30%)/21 (70%)
Affected side (Right/Left)^b^	14 (46.7%)/16 (53.3%)
Height (cm)^a^	159.2 ± 7.4
Weight (kg)^a^	68.0 ± 9.0
Body mass index (kg/m^2^)^a^	26.9 ± 3.0
Preoperative Hip-knee-ankle angle (°)^a^	– 6.5 ± 1.6
Preoperative weight bearing line ratio (%)^a^	21.1 ± 7.4
Postoperative Hip-knee-ankle angle (°)^a^	2.6 ± 1.4
Postoperative weight bearing line ratio (%)^a^	61.1 ± 5.8
Correction angle (°)^a^	10.3 ± 2.2
Preoperative posterior tibial slope (°)^a^	8.9 ± 3.2

^a^The values are presented as mean ± standard deviation.

^b^The values are presented as n (%).

A total of 13 virtual MOWHTO simulations were performed for each 3D model in this study. When the anterior part of the osteotomy line was inclined distally compared to the line parallel to the medial tibial plateau line, the posterior tibial slope increased. As the osteotomy inclination angle increased, the mean change value in the posterior tibial slope also increased. Conversely, when the anterior part of the osteotomy line was inclined proximally compared to the line parallel to the medial tibial plateau line, the posterior tibial slope decreased. As the absolute value of the negative number of posterior inclined osteotomy angle increased, the absolute value of the mean change in the posterior tibial slope also increased further (Table 2). Comparison between the postoperative posterior tibial slopes measured on the 3D models where the anteriorly inclined osteotomy was performed showed statistically significant difference (*p* < 0.001). All pairwise comparisons showed statistically significant differences, except for the comparison of posterior tibial slopes between the simulated 3D models in which the osteotomy was performed with 0° and 5° sagittal osteotomy inclination angle (Table 3). Comparison between the postoperative posterior tibial slopes measured on the 3D models where the posteriorly inclined osteotomy was performed showed statistically significant difference (*p* < 0.001). All pairwise comparisons also showed statistically significant differences, except for the comparison of posterior tibial slopes between the simulated 3D models in which the osteotomy was performed with 0° and − 5° sagittal osteotomy inclination angle (Table 4). Comparison between the postoperative posterior tibial slope after virtual osteotomy with each sagittal osteotomy inclination angle and preoperative posterior tibial slope measured in the original preoperative tibial model showed statistically significant differences, except for the comparison with posterior tibial slopes measured on the simulated 3D models in which the osteotomy was performed with 0°, 5° and − 5° (Table 5).

**Table 2:** Postoperative posterior tibial slopes measured on the 3D models where the virtual osteotomy was performed with various sagittal osteotomy inclination angle.

Sagittal osteotomy inclination angle	Posterior tibial slope	Posterior tibial slope change
30°	11.3 ± 3.6	2.4 ± 0.8
25°	10.9 ± 3.6	2.0 ± 0.8
20°	10.6 ± 3.6	1.7 ± 0.9
15°	10.1 ± 3.5	1.2 ± 0.7
10°	9.6 ± 3.4	0.7 ± 0.7
5°	9.1 ± 3.4	0.2 ± 0.9
0°	8.9 ± 3.3	0.0 ± 0.5
− 5°	8.7 ± 3.3	− 0.2 ± 0.4
− 10°	8.4 ± 3.4	− 0.5 ± 0.5
− 15°	8.2 ± 3.4	− 0.7 ± 0.5
− 20°	7.7 ± 3.4	− 1.2 ± 0.5
− 25°	7.3 ± 3.4	− 1.6 ± 0.6
− 30°	6.9 ± 3.3	− 2.0 ± 0.7

The values are presented as mean ± standard deviation.

**Table 3:** Comparison between the postoperative posterior tibial slopes measured on the 3D models where the anteriorly inclined osteotomy and pairwise comparison.

Posterior tibial slope^a^		0°	5°	10°	15°	20°	25°	30°	*p* value^b^
		8.9 ± 3.3	9.1 ± 3.4	9.6 ± 3.4	10.1 ± 3.5	10.6 ± 3.6	10.9 ± 3.6	11.3 ± 3.6	< 0.001
Pairwise Comparison^c^	0°		> 0.999	< 0.001	< 0.001	< 0.001	< 0.001	< 0.001	–
	5°	> 0.999		< 0.001	< 0.001	< 0.001	< 0.001	< 0.001	–
	10°	< 0.001	< 0.001		0.001	< 0.001	< 0.001	< 0.001	–
	15°	< 0.001	< 0.001	0.001		0.001	< 0.001	< 0.001	–
	20°	< 0.001	< 0.001	< 0.001	0.001		0.002	< 0.001	–
	25°	< 0.001	< 0.001	< 0.001	< 0.001	0.002		0.005	–
	30°	< 0.001	< 0.001	< 0.001	< 0.001	< 0.001	0.005		–

^a^The values are presented as mean ± standard deviation.

^b^Analyzed by repeated measured analysis of variance (ANOVA).

^c^Adjusted *p* value obtained by Bonferroni correction.

**Table 4:** Comparison between the postoperative posterior tibial slopes measured on the 3D models where the posteriorly inclined osteotomy and pairwise comparison.

Posterior tibial slope^a^		0°	− 5°	− 10°	− 15°	− 20°	− 25°	− 30°	*p* value^b^
		8.9 ± 3.3	8.7 ± 3.3	8.4 ± 3.4	8.2 ± 3.4	7.7 ± 3.4	7.3 ± 3.4	6.9 ± 3.3	< 0.001
Pairwise Comparison^c^	0°		> 0.999	0.002	< 0.001	< 0.001	< 0.001	< 0.001	–
	− 5°	> 0.999		0.001	< 0.001	< 0.001	< 0.001	< 0.001	–
	− 10°	0.002	0.001		0.019	< 0.001	< 0.001	< 0.001	–
	− 15°	< 0.001	< 0.001	0.019		< 0.001	< 0.001	< 0.001	–
	− 20°	< 0.001	< 0.001	< 0.001	< 0.001		< 0.001	< 0.001	–
	− 25°	< 0.001	< 0.001	< 0.001	< 0.001	< 0.001		< 0.001	–
	− 30°	< 0.001	< 0.001	< 0.001	< 0.001	< 0.001	< 0.001		–

^a^The values are presented as mean ± standard deviation.

^b^Analyzed by repeated measured analysis of variance (ANOVA).

^c^Adjusted *p* value obtained by Bonferroni correction.

**Table 5:** Comparison between the postoperative posterior tibial slope after virtual osteotomy with each sagittal osteotomy inclination angle and preoperative posterior tibial slope (8.9 ± 3.2°) measured in the original preoperative tibial model.

Sagittal osteotomy inclination angle	− 30°	− 25°	− 20°	− 15°	− 10°	− 5°	0°	5°	10°	15°	20°	25°	30°
Posterior tibal slope^a^	6.9 ± 3.3	7.3 ± 3.4	7.7 ± 3.4	8.2 ± 3.4	8.4 ± 3.4	8.7 ± 3.3	8.9 ± 3.3	9.1 ± 3.4	9.6 ± 3.4	10.1 ± 3.5	10.6 ± 3.6	10.9 ± 3.6	11.3 ± 3.6
*p* value^b^	< 0.001	< 0.001	< 0.001	< 0.001	< 0.001	0.056	0.994	0.281	< 0.001	< 0.001	< 0.001	< 0.001	< 0.001

^a^The values are presented as mean ± standard deviation.

^b^Analyzed by paired *t*-test.

**Figure 6:**
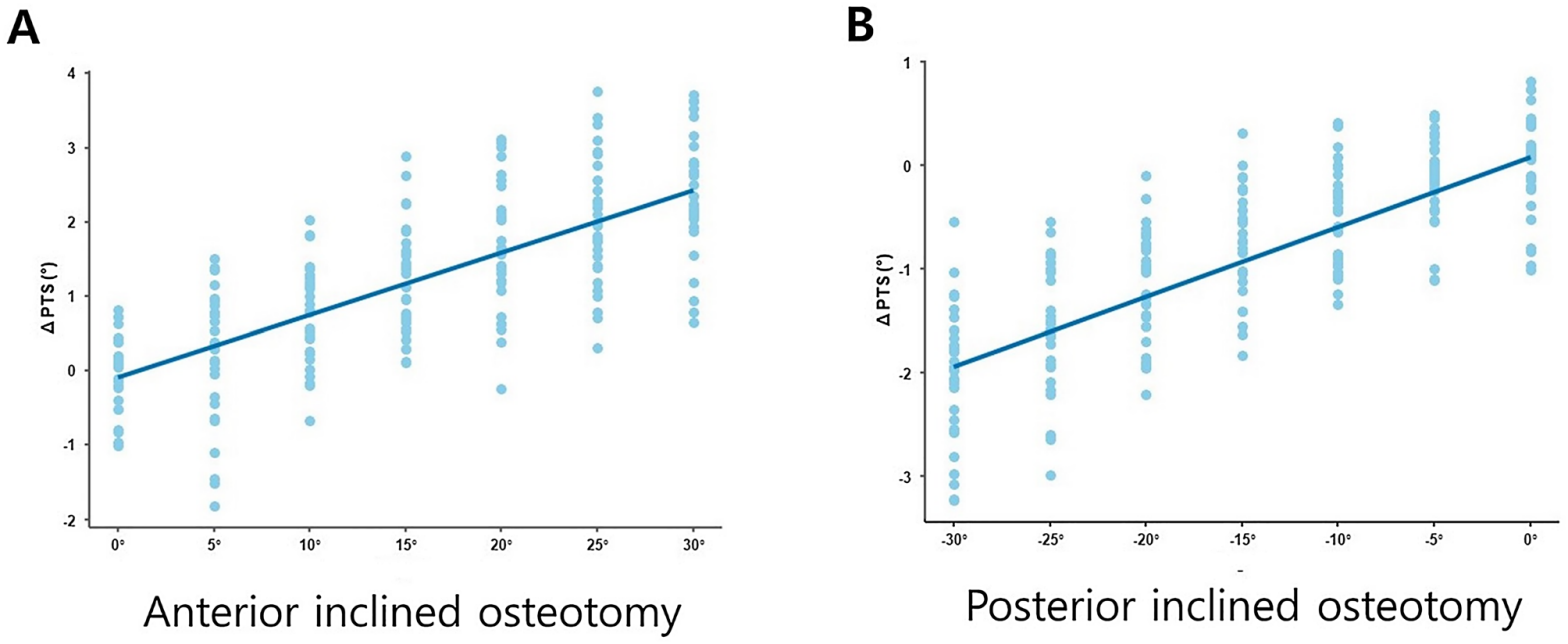
Scatter plots of the posterior tibial slopes according to the change of the sagittal osteotomy inclination angle. (**A**) Anteriorly inclined osteotomy. (**B**) Posteriorly inclined osteotomy. These figures were created using the R statistical software (version 3.6.2; R Foundation for Statistical Computing, Vienna, Austria).

According to the linear mixed model analysis, the sagittal osteotomy inclination angle had a significant influence on the change of the posterior tibial slope in both anteriorly and posteriorly inclined osteotomies (*p* < 0.001). When the sagittal osteotomy inclination angle was positive, an increase in the osteotomy inclination angle by 1° increased the posterior tibial slope by 0.079° (*β* = 0.079, *P* < 0.001). Conversely, when the sagittal osteotomy inclination angle was negative, a 1° decrease in the osteotomy inclination angle decreased the posterior tibial slope by 0.067° (*β* = 0.067, *p* < 0.001) (Table 6). Figure 6 shows the scatter plots of the posterior tibial slopes changes according to the 13 sagittal osteotomy inclination angles. The estimated means with 95% confidence interval obtained from a linear mixed model under the random intercept model also showed that osteotomy inclination angles of greater than + 10° and less than –10° had statistical significant effects on the change in the posterior tibial slope.

**Table 6:** Analysis of the effect of sagittal osteotomy inclination angle on the change in the posterior tibial slope by linear mixed model.

	Anteriorly inclined osteotomy		Posteriorly inclined osteotomy	
	β (S.E)	*p* value	β (S.E)	*p* value
Inclination angle	0.079 (0.006)	< 0.001	0.067 (0.004)	< 0.001

The coefficients were obtained from a linear mixed model under a random intercept model with first order autoregressive structure. S.E. = standard error.

## Discussion

This 3D simulation study was performed to verify how the sagittal osteotomy inclination angle affects the change in the posterior tibial slope and to conduct a quantitative analysis to determine the extent of change in the posterior tibial slope according to the degree of change in the osteotomy inclination angle. After 3D models of preoperative original tibia were reconstructed, 13 osteotomies were performed for each tibial model at intervals of 5° from − 30° to 30° of sagittal osteotomy inclination angle. According to the measurement results of the posterior tibial slope, anteriorly and posteriorly inclined osteotomy caused proportional increase and decrease in the posterior tibial slope, respectively. These findings of the current study present three principal implications. First, when there was an inclination angle occurred between the osteotomy line and the medial tibial plateau line in the sagittal plane, the posterior tibial slope changed after MOWHTO. Second, with respect to the line parallel to the medial tibial plateau line, when the anterior portion of the osteotomy line was inclined distally, the posterior tibial slope increased, and when the anterior portion of the osteotomy line was inclined proximally, the posterior tibial slope decreased. Third, as the absolute value of the osteotomy inclination angle in the sagittal plane increased, the degree of change in the posterior tibial slope also increased.

The changes in the posterior tibial slope were observed after MOWHTO when there was an osteotomy inclination angle with an absolute value of 10° or more between the osteotomy line and the medial tibial plateau line in the sagittal plane. Multiple factors need to be appraised during MOWHTO to prevent unwanted changes in the posterior tibial slope^20–23^. The most common factor to influence the change in the posterior tibial slope is the opening gap ratio, which is derived based on the difference between anterior and posterior osteotomy opening gaps^20,21^. However, the present study demonstrated that even if the osteotomy opening gap ratio is adjusted to an appropriate value of 2/3 during MOWHTO^21^, the postoperative posterior tibial slope can change depending on the osteotomy inclination angle in the sagittal plane. These results are consistent with those of previous clinical studies^22,23^. In addition, some previous studies have also argued that the posterior tibial slope did not change when an osteotomy line was created parallel to the joint line^32,33^. Miller et al.^32^ suggested that making the sagittal osteotomy line parallel to the medial tibial plateau was essential to avoid the change of the posterior tibial slope. Moon et al.^33^ recently reported an experimental simulation study using a 3D square column model, which showed that no significant difference in the posterior tibial slope change was observed when the osteotomy line was parallel to the posterior tibial slope. The results of the present study were consistent with those of the aforementioned studies. In actual MOWHTO surgery, most of the osteotomy lines have been reported to be non-parallel to the medial tibial plateau lines in the sagittal plane^22,23^. Therefore, care should be taken to perform the osteotomy parallel to the medial tibia plateau line in the sagittal plane in order to avoid an unwanted change in the posterior tibial slope.

Furthermore, the findings of the present study revealed that the orientation of the sagittal osteotomy angle affected the increase or decrease in the posterior tibial slope. With respect to the line parallel to the medial tibial plateau, when the anterior portion of the osteotomy line was inclined distally, the posterior tibial slope increased, and when the anterior portion of the osteotomy line was inclined proximally, the posterior tibial slope decreased. Previous studies also have reported on the relationship between the osteotomy inclination angle in the sagittal plane and the change in the posterior tibial slope^22,23^. Lee et al.^22^ noted that 87.1% of the osteotomy lines were inclined anteriorly and the mean osteotomy inclination angle was 15.1° in the sagittal plane. Anteriorly inclined osteotomy in the sagittal plane was related to an increase in the posterior tibial slope. Chung et al.^23^ demonstrated the effect of the anteriorly and posteriorly inclined osteotomy on the posterior tibial slope. The mean increase in the posterior tibial slope was 1° in patients with an anteriorly inclined osteotomy line, whereas the mean decrease in the posterior tibial slope was 0.9° in patients with a posteriorly inclined osteotomy line. The results of the present simulation study were in agreement with those of previous clinical studies. In the present study, 13 virtual osteotomies were performed for each 3D tibial model to systematically analyze the effect of osteotomy line orientation on the change in the posterior tibial slope. One osteotomy was performed parallel to the medial tibial plateau line, while six anteriorly inclined osteotomies and six posteriorly inclined osteotomies were performed intervals of 5°. The results of this study showed that the posterior tibial slope increased in all cases of anteriorly inclined osteotomy and decreased in all cases of posteriorly inclined osteotomy. The virtual osteotomy simulation could exclude the other influencing factors that might affect the change in the posterior tibial slope, such as the osteotomy opening gap ratio, by adjusting the ratio to an appropriate value of 2/3. Therefore, the effect of the sagittal osteotomy inclination angle on the change in the posterior tibial slope could be more clearly elucidated in the present study compared to previous clinical studies^22,23^.

Another notable finding in this study was that, as the absolute value of the sagittal osteotomy inclination angle increased, the degree of change in the posterior tibial slope also increased, regardless of whether the osteotomy line was inclined distally or proximally. According to the results of the linear mixed model analysis, the sagittal osteotomy inclination angle significantly affected the change in the posterior tibial slope in both anterior and posterior osteotomy inclinations, and the change value of the posterior tibial slope increased in proportion to the absolute value of the sagittal osteotomy inclination angle. When the sagittal osteotomy inclination was anterior, if the osteotomy inclination angle increased by 1°, the posterior tibial slope also increased by 0.079° (*β* = 0.079, *p* < 0.001). Conversely, when the sagittal osteotomy inclination was posterior, if the osteotomy inclination angle decreased by 1°, the posterior tibial slope also decreased by 0.067° (*β* = 0.067, *p* < 0.001). In addition, another noteworthy finding was that when the absolute value of the sagittal osteotomy inclination angle was ≥ 10°, the posterior tibial slope changed significantly before and after osteotomy. According to a previous study^34^, the mean sagittal osteotomy plane angle in 3D CT was 6.2° anteriorly, and this angle did not significantly affect the change in the posterior tibial slope. The results of this previous study differed from those of the present study in that there was no significant difference in the posterior tibial slope after osteotomy, although the sagittal osteotomy angle had an anterior inclination. However, the mean value of the sagittal osteotomy inclination angle in the previous study was 6.2°, which was less than 10°. Even in the present study, significant change appeared only at the angles of 10° or above, in agreement with Akamatsu et al.^34^ To prevent an unintentional change in the posterior tibial slope, it is necessary to make the osteotomy line as parallel to the medial tibial plateau line as possible in the sagittal plane. Conversely, a change in the posterior tibial slope can be clinically used for effective treatment. If a change in the posterior tibial slope is required, the desired change can be achieved by determining the orientation and specific value of the sagittal osteotomy inclination angle during osteotomy, considering the numerical relationship between the sagittal osteotomy inclination angle and change in the posterior tibial slope. Increasing the posterior tibial slope would be beneficial in posterior cruciate ligament-deficient knee and genu recurvatum^15,35,36^, whereas decreasing the posterior tibial slope would be helpful in anterior cruciate ligament-deficient knee^37^.

The present study had several limitations. First, this study was conducted by simulation using virtual osteotomy with a 3D reconstructed CT model. This might be different from the actual osteotomy due to possible anatomical variations of the proximal tibia and adjacent soft tissues, such as the pes anserinus or medial collateral ligament. However, simulation study can offer the advantage of performing several osteotomies on a single tibia model. Second, the sagittal osteotomy inclination angles were set at intervals of 5° rather than continuous values. Thus, an accurate cut-off value of the sagittal osteotomy inclination angle that did not affect the change in the posterior tibial slope could not be obtained. Third, P2, which was used as a hinge, was located 1.0 cm medial to the lateral cortex of the tibia, and 1.5 cm below the articular surface in the coronal plane. However, there may be some individual differences of hinge location. Fourth, the observed change value in the posterior tibial slope was not large. Even when an osteotomy angle of 30° was given, the mean change in the posterior tibial slope was 2.4°, and a − 30° osteotomy angle resulted in a mean change of − 2.0° in the posterior tibial slope. The maximum change in the value of the posterior tibial slope was 3.72°. It has not yet been reported to what extent changes in the posterior tibia slope actually have a significant effect on the biomechanical aspect of the knee. However, previous studies have reported that even a small change in the posterior tibial slope affects the load on the anterior cruciate ligament^38,39^. Further research is needed for an in-depth elucidation of the clinical significance of the change in the posterior tibial slope in relation to the sagittal osteotomy inclination angle.

## Conclusions

The osteotomy inclination angle in the sagittal plane significantly affected the posterior tibial slope. When there was an inclination angle occurred between the osteotomy line and the medial tibial plateau line in the sagittal plane, the posterior tibial slope changed after MOWHTO. The posterior tibial slope tended to increase in anteriorly inclined osteotomy and decrease in posteriorly inclined osteotomy. The change in the posterior tibial slope was proportionally related to the absolute value of the osteotomy inclination angle. This study can help prevent unintentional changes in the posterior tibial slope during MOWHTO by predicting the changes in posterior tibial slope according to the sagittal osteotomy inclination angle.

## Supplementary Information

Supplementary Information.

## Data Availability

The datasets generated and analyzed in the current study are not publicly available to protect the patients’ personal information, but are available from the corresponding author on reasonable request.
